# Significant changes in synovial fluid microRNAs after high tibial osteotomy in medial compartmental knee osteoarthritis: Identification of potential prognostic biomarkers

**DOI:** 10.1371/journal.pone.0227596

**Published:** 2020-01-10

**Authors:** Yoon Hae Kwak, Dae-Kyung Kwak, Nan Young Kim, Yun Joong Kim, Jeong Seop Lim, Je-Hyun Yoo

**Affiliations:** 1 Department of Orthopaedic Surgery, Severance Children’s Hospital, Yonsei University College of Medicine, Seoul, Republic of Korea; 2 Department of Orthopaedic Surgery, Hallym University Sacred Heart Hospital, Hallym University College of Medicine, Anyang, Republic of Korea; 3 Hallym Institute of Translational Genomics & Bioinformatics, Hallym University Medical Center, Anyang, Republic of Korea; 4 Ilsong Institute of Life Science, Hallym University, Anyang, Republic of Korea; 5 Department of Neurology, Hallym University Sacred Heart Hospital, Hallym University College of Medicine, Anyang, Republic of Korea; Katholieke Universiteit Leuven Rega Institute for Medical Research, BELGIUM

## Abstract

High tibial osteotomy (HTO) is a well-established treatment for medial compartmental knee osteoarthritis. Several microRNAs (miRNAs) are involved in osteoarthritis progression and are useful as osteoarthritis-related biomarkers. In this prospective study, we investigated differentially expressed microRNAs in the synovial fluid (SF) before and after HTO in patients with medial compartmental knee osteoarthritis to identify microRNAs that can be used as prognostic biomarkers. We used miRNA-PCR arrays to screen for miRNAs in SF samples obtained preoperatively and 6 months postoperatively from 6 patients with medial compartmental knee osteoarthritis who were treated with medial open wedge HTO. Differentially expressed miRNAs identified in the profiling stage were validated by real-time quantitative PCR in 22 other patients who had also been treated with HTO. All patients radiographically corresponded to Kellgren-Lawrence grade II or III with medial compartmental osteoarthritis. These patients were clinically assessed using a visual analogue scale and Western Ontario McMaster Universities scores. Mechanical axis changes were measured on standing anteroposterior radiographs of the lower limbs assessed preoperatively and at 6 months postoperatively. Among 84 miRNAs known to be involved in the inflammatory process, 14 were expressed in all SF specimens and 3 (miR-30a-5p, miR-29a-3p, and miR-30c-5p) were differentially expressed in the profiling stage. These 3 miRNAs, as well as 4 other miRNAs (miR-378a-5p, miR-140-3p, miR-23a-3p, miR-27b-3p), are related to osteoarthritis progression. These results were validated in the SF from 22 patients. Clinical and radiological outcomes improved after HTO in all patients, and only 2 miRNAs (miR-30c-5p and miR-23a-3p) were significantly differentially expressed between preoperative and postoperative 6-month SF samples (p = 0.006 and 0.007, respectively). Of these two miRNAs, miR-30c-5p correlated with postoperative pain relief. This study provides potential prognostic miRNAs after HTO and further investigations should be considered to determine clinical implications of these miRNAs.

## Introduction

High tibial osteotomy (HTO) is a well-known treatment modality for active patients with medial compartmental osteoarthritis (OA) of the knee [1,2]. Several studies have shown a correlation between HTO and regeneration of the degenerated cartilage, which is thought to occur through the realignment of the mechanical axis in medial compartmental knee OA. As a result, HTO offers excellent intermediate and long-term survival rates [3,4]. Moreover, a correlation has been reported between visible improvement of the articular surface and the degree of correction with HTO [5].

The pathogenesis of OA is thought to be a complex and multifactorial process caused by genetic, mechanical, and environmental factors. Recently, the effect of dysregulation, which occurs at the molecular level during the pathogenic process of OA, has gained increased attention [6]. Environmental factors induce epigenetic mechanisms that independently regulate genomic activity in the DNA sequence and alter the expression of genes involved in disease development [7]. MicroRNAs (miRNAs), which are small non-coding RNAs composed of 19–25 nucleotides, regulate gene expression post-transcriptionally through mRNA degradation or translational suppression by binding to the 3ʹ-UTR of target genes [8]. The role of miRNAs in cartilage degeneration has been previously reported [9,10]. Most published studies that quantified miRNA expression in OA have focused on tissues or cell lines. However, miRNAs have also been identified in the circulatory body fluids of patients with other pathologies [11,12] such that the levels of some circulating miRNAs have been associated with the diagnosis and prognosis of several diseases [13].

Most studies on HTO in medial compartmental knee OA have focused on the change in the mechanical load on the knee joint resulting from the realignment of the mechanical axis (MA) [14,15]. Regeneration of the degenerated articular cartilage in the medial compartment in medial compartmental knee OA can be expected following HTO without the need for any additional procedures, although the detailed mechanism is still unclear. Although there are many studies related to HTO in medial compartmental knee OA or miRNA expression in OA, only a small amount of information is available on the changes that occur in miRNA expression after HTO in medial compartmental knee OA and their clinical implication. Nonetheless, any changes in miRNA expression in the synovial fluid (SF) could be related to the change in the MA following HTO and subsequent cartilage regeneration. Thus, these miRNAs could be used as biomarkers to predict the prognosis after HTO.

This prospective study was conducted to determine which miRNAs in the SF are significantly differentially expressed after HTO for medial compartment knee OA and to identify the miRNAs that can serve as biomarkers related to overall prognosis.

## Materials and methods

### Study subjects

Patients with medial compartment knee OA who underwent HTO between November 2015 and February 2017 were prospectively enrolled in this study. First, 6 patients were recruited for the profiling stage; these patients were all females with a mean age of 57.7 years (52–65 years). Medial compartmental knee OA was confirmed by plain radiographs and scored according to the Kellgren-Lawrence grade (K-L grade) (Table 1). All patients were K-L grade II or III OA only in the medial compartment of the affected knee. For miRNA validation, 22 patients were recruited, including 19 female and 3 male patients with a mean age of 56.8 years (46–65 years). All patients were K-L grade II or III OA only in the medial compartment (Table 2). SF specimens were collected from each patient in the operating room immediately prior to undergoing the HTO procedure. Six-month SF specimens were collected in an outpatient clinic at the 6-month follow-up postoperatively.

**Table 1:** Data of 6 patients at the profiling stage.

Serial number	Gender	Age (years)	Body mass index (kg/m^2^)	Kellgren-Lawrence grade	Visual Analogue Scale (VAS)	WOMAC Score			
						Total	Pain	Stiffness	Function
1	F	57	28.4	II	6	54	13	4	37
2	F	55	23.9	II	5	10	1	0	9
3	F	61	22.8	III	7	56	8	6	42
4	F	56	27.9	III	7	42	9	3	30
5	F	65	30.2	III	3	14	0	0	14
6	F	52	26.0	III	4	33	9	3	21

**Table 2:** Demographic data of 22 patients at the validation stage.

Serial number	Gender	Age (years)	Body mass index (kg/m^2^)	Kellgren-Lawrence grade	Visual Analogue Scale (VAS)	WOMAC Score			
						Total	Pain	Stiffness	Function
1	F	50	30.5	III	3	21	2	2	17
2	F	46	33.3	III	7	22	3	2	17
3	F	51	25.5	II	9	59	14	3	42
4	F	56	21.8	III	8	28	5	5	18
5	F	57	28.4	II	6	54	13	4	37
6	F	55	23.9	II	5	10	1	0	9
7	F	61	22.8	III	7	56	8	6	42
8	F	56	27.9	III	7	42	9	3	30

Clinical evaluations to determine factors such as pain and functional improvement of the patients were performed using a visual analogue scale (VAS) score and the Western Ontario McMaster Universities (WOMAC) score, both preoperatively and 6 months postoperatively. Radiological assessment was performed using standing full-length anteroposterior radiographs of the lower limbs, which were taken preoperatively and 6 months postoperatively in all cases. These radiographs were taken to ensure that the patella was directly facing the front. Radiographic changes after HTO were evaluated by measuring the MA and weight bearing line (WBL) ratio. The WBL was defined as the perpendicular distance from the WBL to the medial edge of the tibial plateau divided by the width of the tibial plateau. The MA was defined as the angle subtended by a line drawn from the center of the femoral head to the center of the knee and a line drawn from the center of the knee to the center of the talus. The WBL was drawn from the center of the femoral head to the center of the superior articular surface of the talus [16].

### Sampling strategy and miRNA analysis

#### RNA extraction from SF

First, 5 ml of SF was collected in EDTA tubes from all patients, and the plasma was separated by centrifugation within 4 h of collection to ensure consistent pre-analytical conditions for all samples. All samples were stored at -80°C and were not subjected to any freeze–thaw cycles before use. Total RNA was isolated from each specimen using the miRNeasy Serum/Plasma Kit (Qiagen, Germany), according to the manufacturer’s instructions. The samples were immediately stored at -80°C until further analysis.

#### Profiling by miRNAs array analysis

**Fig 1:**
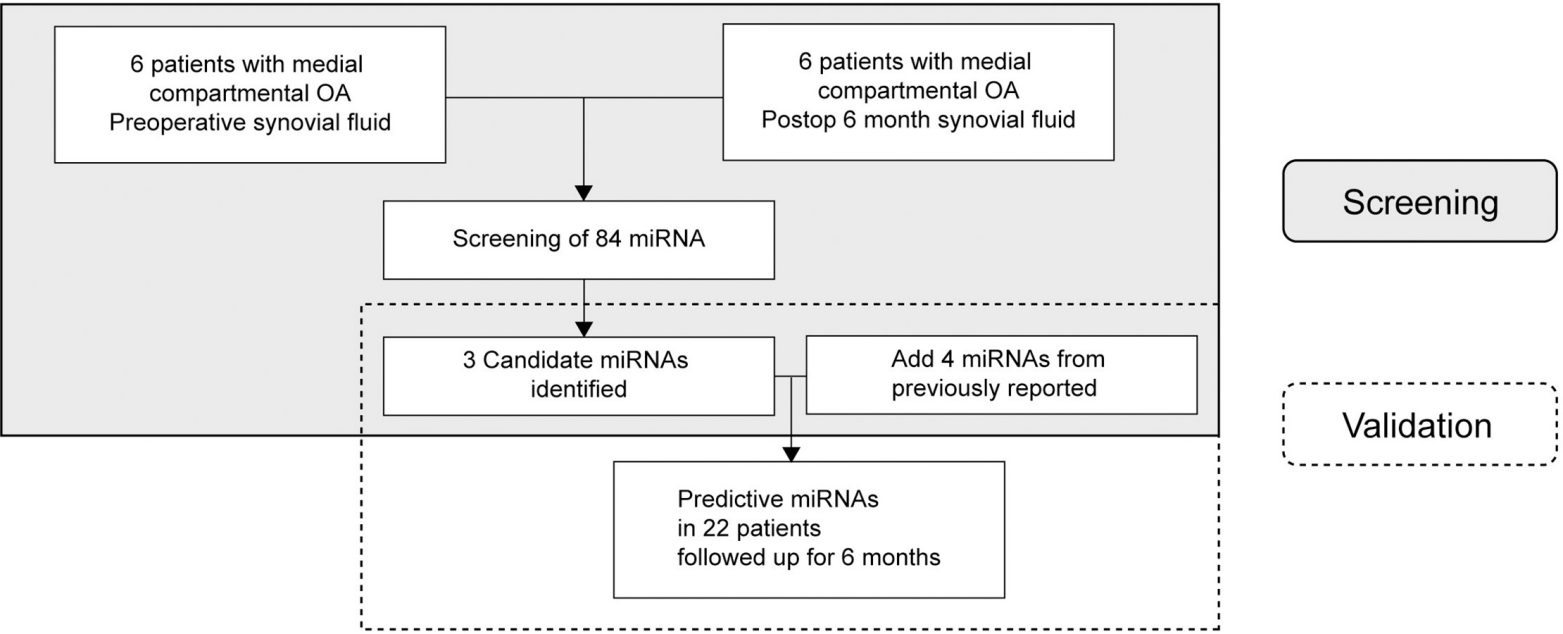
Study design. Three candidate miRNAs were identified in synovial fluid samples collected from 6 patients preoperatively and postoperatively after high tibial osteotomy. After adding 4 miRNAs from published literature, a total of 7 miRNAs in 22 patients were validated.

Reverse transcription was conducted with 2 μl RNA in 10 μl reactions using the miScript II RT Kit (Qiagen, Germany), according to the manufacturer’s instructions. cDNA from miRNA was amplified using the miScript miRNA PCR Array (MIHS-105Z, Qiagen, Germany). Real-time PCR was performed on the StepOnePlus^TM^ Real Time PCR System (Applied Biosystems, USA) using the miScript SYBR Green PCR Kit (Qiagen, Germany). Thermal cycling conditions were 95°C for 15 min followed by 40 cycles of 95°C for 15 s, 55°C for 30 s, and 70°C for 30 s, respectively. The data were analyzed using the PCR array data analysis tools (Qiagen, Germany). These panels contained primers for detection of the 84 most highly expressed miRNAs in the human body fluid (Fig 1). Real-time PCR was performed according to the manufacturer’s instructions. In each 96-well plate, cDNAs from preoperative and postoperative 6-month SF specimens from each patient were amplified in parallel. Expression values were calculated using the 2^-ΔCt^ method, and the mean and standard deviation (SD) of the three technical replicates are shown in Table 3. Additionally, the fold changes indicating the value of the change in the expression of miRNAs between the preoperative and postoperative 6-month time points were also calculated.

**Table 3:** miRNA expression levels in synovial fluid.

miRNA	Preop.		Postop. 6 months		Fold Regulation	p-value
	ΔCt	2^-ΔCt^	ΔCt	2^-ΔCt^		
**hsa-miR-30a-5p**	0.48 ± 0.71	0.72	0.71 ± 1.00	1.58	2.20	0.028*
**hsa-miR-29a-3p**	0.33 ± 1.36	0.80	1.36 ± 1.15	2.47	3.09	0.037*
**hsa-miR-30c-5p**	0.84 ±0.80	0.56	0.80 ± 1.49	1.25	2.23	0.047*
hsa-miR-144-3p	1.72 ± 1.33	0.30	1.33 ± 0.63	0.77	2.53	0.052
hsa-miR-195-5p	-1.58 ± 0.89	3.00	0.89 ± 0.80	1.20	-2.49	0.058
hsa-let-7c-5p	-0.44 ± 1.64	1.35	1.64 ± 1.14	5.36	3.96	0.058
hsa-let-7e-5p	0.90 ± 1.51	0.54	1.51 ± 1.04	1.39	2.59	0.061
hsa-miR-16-5p	-2.19 ± 1.36	4.57	1.36 ± 0.62	1.46	-3.14	0.062
hsa-miR-543	3.08 ± 2.40	0.12	2.40 ± 1.52	0.59	5.00	0.066
hsa-miR-98-5p	3.08 ± 2.40	0.12	2.40 ± 1.77	0.55	4.65	0.071
hsa-miR-1324	3.08 ± 2.40	0.12	2.40 ± 1.83	0.54	4.56	0.072
hsa-miR-211-5p	3.08 ± 2.40	0.12	2.40 ± 1.83	0.54	4.56	0.072
hsa-miR-300	3.08 ± 2.40	0.12	2.40 ± 1.83	0.54	4.56	0.072
hsa-miR-301a-3p	3.08 ± 2.40	0.12	2.40 ± 1.83	0.54	4.56	0.072
hsa-miR-301b-3p	3.08 ± 2.40	0.12	2.40 ± 1.83	0.54	4.56	0.072
hsa-miR-302a-3p	3.08 ± 2.40	0.12	2.40 ± 1.83	0.54	4.56	0.072
hsa-miR-302b-3p	3.08 ± 2.40	0.12	2.40 ± 1.83	0.54	4.56	0.072
hsa-miR-302c-3p	3.08 ± 2.40	0.12	2.40 ± 1.83	0.54	4.56	0.072
hsa-miR-340-5p	3.08 ± 2.40	0.12	2.40 ± 1.83	0.54	4.56	0.072
hsa-miR-34c-5p	3.08 ± 2.40	0.12	2.40 ± 1.83	0.54	4.56	0.072
hsa-miR-372-3p	3.08 ± 2.40	0.12	2.40 ± 1.83	0.54	4.56	0.072
hsa-miR-373-3p	3.08 ± 2.40	0.12	2.40 ± 1.83	0.54	4.56	0.072
hsa-miR-381-3p	3.08 ± 2.40	0.12	2.40 ± 1.83	0.54	4.56	0.072
hsa-miR-410-3p	3.08 ± 2.40	0.12	2.40 ± 1.83	0.54	4.56	0.072
hsa-miR-449b-5p	3.08 ± 2.40	0.12	2.40 ± 1.83	0.54	4.56	0.072
hsa-miR-511-5p	3.08 ± 2.40	0.12	2.40 ± 1.83	0.54	4.56	0.072
hsa-miR-513b-5p	3.08 ± 2.40	0.12	2.40 ± 1.83	0.54	4.56	0.072
hsa-miR-519c-3p	3.08 ± 2.40	0.12	2.40 ± 1.83	0.54	4.56	0.072
hsa-miR-519d-3p	3.08 ± 2.40	0.12	2.40 ± 1.83	0.54	4.56	0.072
hsa-miR-520d-3p	3.08 ± 2.40	0.12	2.40 ± 1.83	0.54	4.56	0.072
hsa-miR-520e	3.08 ± 2.40	0.12	2.40 ± 1.83	0.54	4.56	0.072
hsa-miR-524-5p	3.08 ± 2.40	0.12	2.40 ± 1.83	0.54	4.56	0.072
hsa-miR-545-3p	3.08 ± 2.40	0.12	2.40 ± 1.83	0.54	4.56	0.072
hsa-miR-548c-3p	3.08 ± 2.40	0.12	2.40 ± 1.83	0.54	4.56	0.072
hsa-miR-548d-3p	3.08 ± 2.40	0.12	2.40 ± 1.83	0.54	4.56	0.072
hsa-miR-548e-3p	3.08 ± 2.40	0.12	2.40 ± 1.83	0.54	4.56	0.072
hsa-miR-590-5p	3.08 ± 2.40	0.12	2.40 ± 1.83	0.54	4.56	0.072
hsa-miR-607	3.08 ± 2.40	0.12	2.40 ± 1.83	0.54	4.56	0.072
hsa-miR-655-3p	3.08 ± 2.40	0.12	2.40 ± 1.83	0.54	4.56	0.072
hsa-miR-875-3p	3.08 ± 2.40	0.12	2.40 ± 1.83	0.54	4.56	0.072
hsa-miR-9-5p	3.08 ± 2.40	0.12	2.40 ± 1.83	0.54	4.56	0.072
hsa-miR-454-3p	3.01 ± 2.31	0.12	2.31 ± 1.83	0.54	4.34	0.072
hsa-miR-128-3p	2.84 ± 2.15	0.14	2.15 ± 1.83	0.54	3.88	0.074
hsa-miR-497-5p	2.98 ± 2.40	0.13	2.40 ± 1.83	0.54	4.26	0.075
hsa-miR-449a	3.07 ± 2.40	0.12	2.40 ± 1.98	0.62	5.20	0.076
hsa-miR-101-3p	2.53 ± 1.93	0.17	1.93 ± 1.68	0.56	3.26	0.077
hsa-miR-374a-5p	2.69 ± 2.18	0.16	2.18 ± 1.83	0.54	3.48	0.080
hsa-miR-17-5p	1.26 ± 0.79	0.42	0.79 ± 0.76	0.74	1.77	0.082
hsa-miR-34a-5p	2.15 ± 1.97	0.23	1.97 ± 1.69	0.61	2.73	0.084
hsa-miR-20b-5p	1.91 ± 1.30	0.27	1.30 ± 1.80	0.57	2.15	0.085
hsa-miR-181d-5p	2.21 ± 1.62	0.22	1.62 ± 1.83	0.54	2.50	0.091
hsa-miR-424-5p	1.92 ± 1.29	0.26	1.29 ± 1.69	0.56	2.13	0.093
hsa-miR-130a-3p	2.03 ± 1.45	0.24	1.45 ± 1.83	0.54	2.21	0.094
hsa-miR-202-3p	2.44 ± 2.04	0.18	2.04 ± 1.89	0.60	3.26	0.098
hsa-miR-106b-5p	1.99 ± 1.50	0.25	1.50 ± 1.83	0.54	2.15	0.099
hsa-miR-125b-5p	-0.71 ± 1.90	1.63	1.90 ± 0.88	5.40	3.31	0.101
hsa-miR-145-5p	2.64 ± 2.16	0.16	2.16 ± 1.88	0.57	3.57	0.104
hsa-miR-30b-5p	2.25 ± 2.18	0.21	2.18 ± 1.41	0.61	2.90	0.108
hsa-miR-181c-5p	1.94 ± 1.69	0.26	1.69 ± 1.83	0.54	2.07	0.135
hsa-miR-130b-3p	1.60 ± 1.25	0.33	1.25 ± 1.59	0.58	1.75	0.138
hsa-let-7g-5p	1.52 ± 1.42	0.35	1.42 ± 1.30	0.74	2.13	0.140
hsa-miR-656-3p	0.90 ± 2.50	0.53	2.50 ± 1.94	2.35	4.39	0.147
hsa-miR-186-5p	0.96 ± 0.60	0.51	0.60 ± 0.98	0.73	1.42	0.150
hsa-miR-125a-5p	-0.55 ± 1.19	1.46	1.19 ± 1.34	3.24	2.22	0.169
hsa-miR-181b-5p	-0.25 ± 0.91	1.19	0.91 ± 0.62	0.77	-1.55	0.173
hsa-miR-19a-3p	1.04 ± 0.99	0.49	0.99 ± 1.24	0.68	1.40	0.231
hsa-miR-181a-5p	0.99 ± 0.74	0.51	0.74 ± 1.17	0.65	1.29	0.235
hsa-let-7a-5p	-0.28 ± 1.40	1.22	1.40 ± 1.30	2.17	1.78	0.237
hsa-miR-29c-3p	0.79 ± 1.49	0.58	1.49 ± 2.10	0.74	1.28	0.276
hsa-miR-23a-3p	-3.45 ± **1.92**	10.95	1.92 ± 2.19	18.13	1.65	0.310
hsa-miR-93-5p	1.09 ± 1.21	0.47	1.21 ± 1.02	0.68	1.45	0.343
hsa-miR-29b-3p	2.03 ± 1.81	0.24	1.81 ± 1.53	0.59	2.41	0.351
hsa-let-7f-5p	0.26 ± 0.94	0.84	0.94 ± 1.02	1.16	1.38	0.355
hsa-let-7i-5p	0.95 ± 0.88	0.52	0.88 ± 1.83	0.54	1.04	0.361
hsa-miR-23b-3p	0.18 ± 1.91	0.88	1.91 ± 1.30	1.67	1.89	0.434
hsa-miR-15a-5p	1.55 ± 1.62	0.34	1.62 ± 1.83	0.54	1.58	0.446
hsa-miR-30e-5p	0.22 ± 1.13	0.86	1.13 ± 1.49	1.01	1.18	0.481
hsa-miR-19b-3p	1.34 ± 1.49	0.39	1.49 ± 1.59	0.58	1.47	0.594
hsa-let-7b-5p	-2.92 ± 2.01	7.58	2.01 ± 0.99	11.19	1.48	0.782
hsa-miR-15b-5p	0.27 ± 0.85	0.83	0.85 ± 0.76	0.82	-1.01	0.841
hsa-miR-20a-5p	0.96 ± 1.71	0.51	1.71 ± 0.66	0.76	1.48	0.845
hsa-miR-21-5p	-4.44 ± 1.01	21.75	1.01 ± 0.80	22.94	1.05	0.952
hsa-let-7d-5p	0.51 ± 1.54	0.70	1.54 ± 1.07	0.87	1.24	0.992
hsa-miR-30d-5p	-0.30 ± 1.11	1.23	1.11 ± 0.92	1.34	1.08	0.999

Values are given as the mean ± standard deviation.

*p-value < 0.05

#### Validation RT-qPCR

To analyze the expression of these miRNAs, real-time PCRs were performed using the specific miScript Primer Assay (Qiagen, Germany). To normalize the expression of the miRNAs being validated, the GeNorm algorithm (GenEx, Qiagen, Germany) was used. *SNORD61* was used as an endogenous reference gene [17].

### Ethics statement

This study protocol was approved by the local Institutional Review Board/ Ethics Committee of Hallym University Sacred Heart Hospital (2015-I101), and it was conducted in compliance with the Declaration of Helsinki. Written informed consent was obtained from each patient, and this study was supported by a grant from the National Research Foundation of Korea (NRF-2015R1D1A1A01060157). Personally identifiable information of patients was encrypted and all the analyzed data were anonymized.

### Statistical analysis

Statistical analyses were performed using SPSS^®^ software version 23 (IBM Corporation, Armonk, NY, USA). Paired t-tests for the levels of miRNAs (^Δ^Ct) and a nominal p-value < 0.05 were used in the profiling stage, and Bonferroni corrected p-values (0.05/7) were used in the validation stage. To compare the changes in postoperative miRNAs levels with clinical and radiologic outcomes, correlation analysis using the Spearman rank test was performed considering covariates, including age, gender, BMI, preoperative K-L grade, and preoperative lower leg alignment. Wilcoxon signed-rank test was used for the comparison of clinical outcomes between preoperative and postoperative values.

## Results

### Identification of differentially expressed miRNAs in the profiling stage

**Fig 2:**
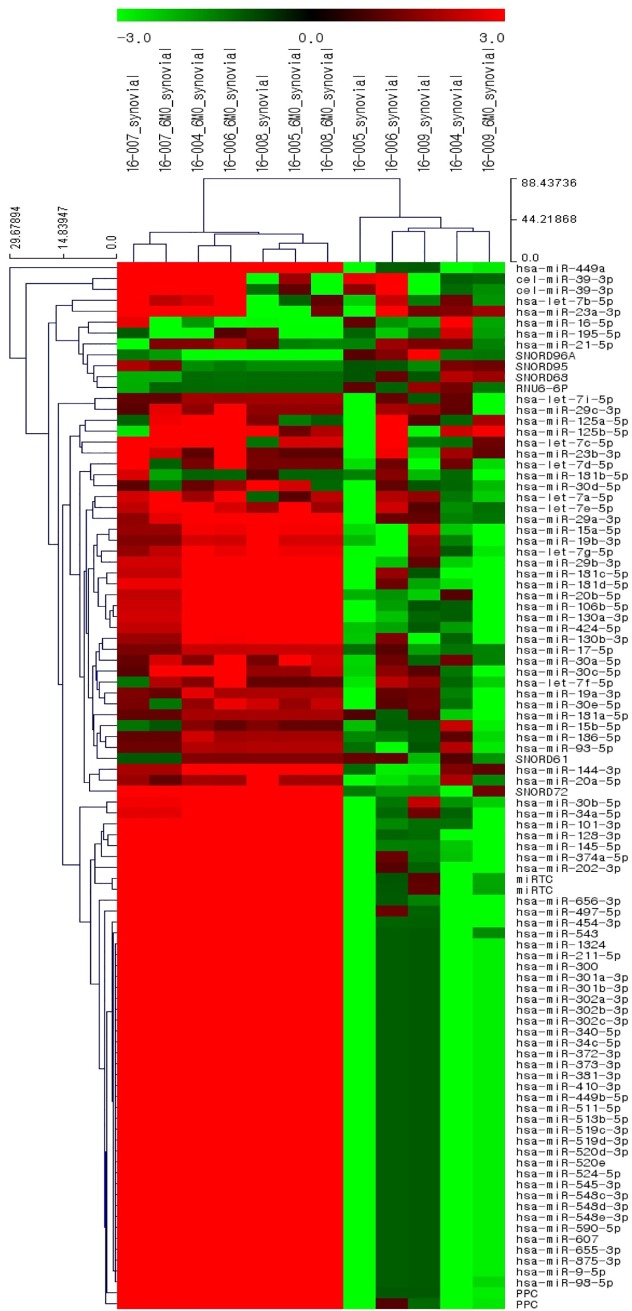
Heat map showing the expression levels of 84 target miRNAs from a miRNA PCR array screening of synovial fluid collected from 6 patients preoperatively and at 6 months postoperatively.

A panel of 84 miRNAs and controls were profiled in SF specimens from 6 patients with medial compartment OA. The expression results for the 84 miRNAs based on biological groups are shown in a heat map (Fig 2), and the PCR array analysis revealed 14 of the 84 target miRNAs in all SF specimens. Only 3 miRNAs, miR-30a-5p, miR-29a-3p, and miR-30c-5p, were upregulated with a significant fold change, and these were included in the validation testing (Table 3).

### Validation stage: Expression of 7 miRNAs in SF samples acquired preoperatively and 6 months postoperatively in 22 patients who underwent HTO

**Fig 3:**
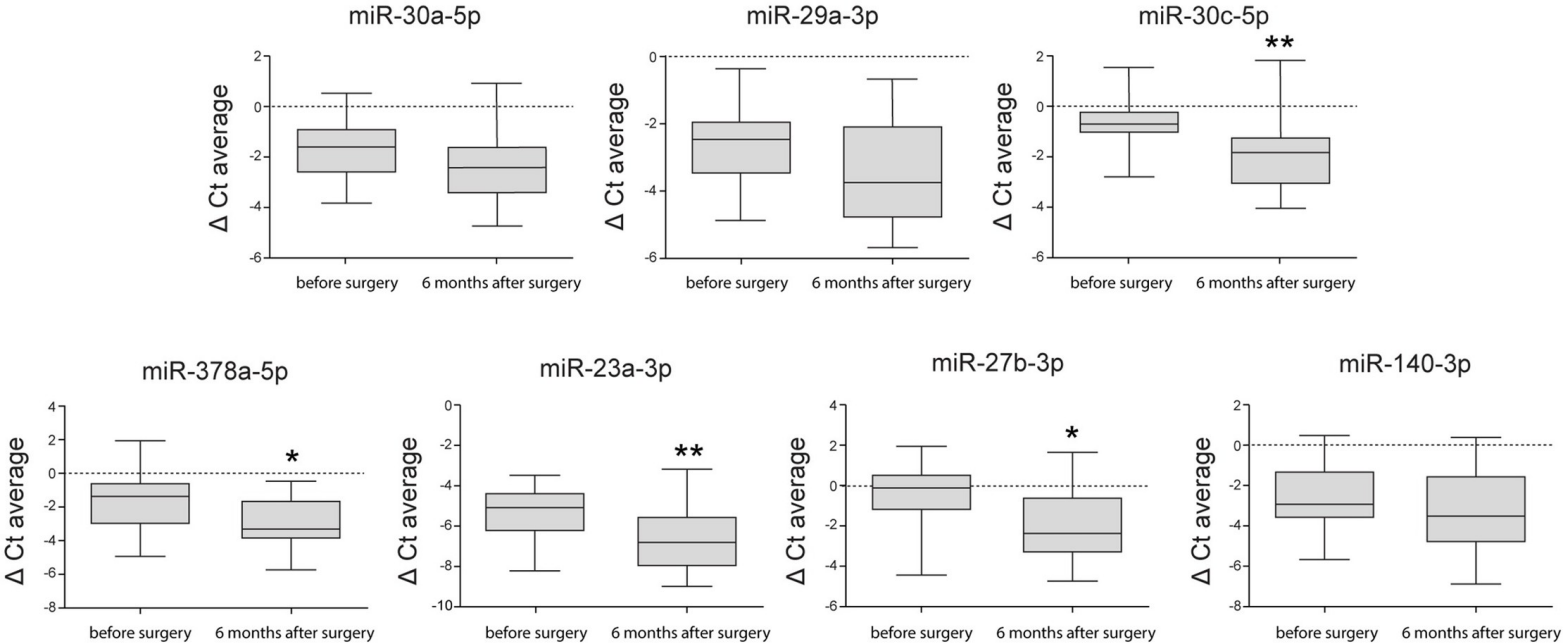
Box plot showing the interquartile range and median of expression levels of miR-30a-5p, miR-29a-3p, miR-30c-5p, miR-378a-5p, miR-23a-3p, miR-27b-3p, and miR-140-3p at the validation stage. The values represent the relative levels normalized to the levels of the reference gene *SNORD61*.

To further validate our profiling results, the expression of a total of 7 miRNAs, the 3 identified in the profiling stage and 4 other miRNAs, miR-378a-5p, miR-140-3p, miR-23a-3p, and miR-27b-3p, which are reported to be related to the progression of OA, were measured in the SF that was acquired preoperatively and at 6 months postoperatively in 22 patients using RT-qPCR analysis. Of the 7 miRNAs, 4 miRNAs, miR-30c-5p, miR-378a-5p, miR-23a-3p and miR-27b-3p, were differentially expressed preoperatively and 6 months postoperatively when normalized to the expression of *SNORD61* (Fig 3). When the p-values were corrected by the Bonferroni method, only 2 miRNAs, miR-30c-5p and miR-23a-3p, showed a significantly positive fold change (Table 4).

**Table 4:** Fold changes of the expression of miRNAs at the profiling and validation stages.

	Profiling stage using microarray		Validation stage using real-time PCR	
miR Name	Fold change	p-value	Fold change	p-value
has-miR-30a-5p	2.20	0.028	2.65	0.101
has-miR-29a-3p	3.09	0.037	3.59	0.062
**has-miR-30c-5p**	2.23	0.047	3.59	0.007*
has-miR-378a-5p	-	-	4.92	0.023
**has-miR-23a-3p**	-	-	4.90	0.006*
has-miR-27b-3p	-	-	6.16	0.012
has-miR-140-3p	-	-	2.65	0.101

*Paired t-test. A Bonferroni corrected p-value < 0.0071 (0.05/7), which is marked with an asterisk, was considered statistically significant.

### Comparison of clinical and radiological outcomes preoperatively and 6 months postoperatively in 22 patients

Clinical outcomes assessed using a VAS and WOMAC questionnaire were significantly improved 6 months postoperatively compared to the preoperative values (Table 5).

**Table 5:** Improvement of radiological and functional outcomes in 22 patients.

	Preoperative	Postoperative 6-month	p-value*
Mechanical axis	-5.4 ± 2.3	+2.6 ± 1.7	<0.001
(-: varus, +: valgus)			
Weight-bearing line ratio (%)	18.6 ± 7.2	59.5 ± 9.2	<0.001
VAS	6.0 ± 2.0	2.6 ± 1.2	<0.001
WOMAC Total	38.9 ± 15.1	24.3 ± 12.0	0.001
Pain	8.0 ± 4.2	4.5 ± 2.6	0.003
Stiffness	3.4 ± 1.8	2.0 ± 1.5	0.008
Function	27.4 ± 10.3	17.8 ± 9.2	0.001

*p-value < 0.05

Preoperative pain, functional disability, and lower leg varus knee alignment were significantly improved 6 months after index surgery in 22 patients.

### Correlation between demographic and clinical data and the expression of miRNAs

There was no significant correlation between demographic data, including age, gender, and body mass index, and the expression of the 7 miRNAs. Moreover, there was no significant correlation between the extent of radiologic correction and expression of the 7 miRNAs investigated. Only the extent of pain on the VAS scale at 6 months postoperatively was significantly lower along with the increased expression of 4 miRNAs [miR-30c-5p (correlation coefficient -0.453, p = 0.034), 27b-3p (correlation coefficient -0.449, p = 0.036), 140-3p (correlation coefficient -0.489, p = 0.021), and 30a-5p (correlation coefficient -0.480, p = 0.024)] (S1 Table). miR-30c-5p was the only biomarker that correlated with significant fold changes after Bonferroni correction and clinical symptoms such as postoperative pain.

## Discussion

Even with pre-existing arthritis in the medial compartment of the knee, many studies have shown improvement of articular cartilage after HTO [5,18]. Second-look arthroscopic assessment after HTO has been used to provide evidence of cartilage regeneration and mechanical improvement, which proves the effectiveness of the operation on cartilage regeneration, although the underlying mechanism is unknown. Advanced techniques using novel circulating miRNA signatures have led to the use of miRNAs as promising biomarkers. These miRNAs were previously shown to be either diagnostic factors or underlying causative factors [19, 20].

Our study was designed to identify SF miRNAs in patients with improved medial compartment OA using a comprehensive array approach. A systemic, array-based miRNA analysis was performed on SF samples from 6 patients preoperatively and postoperatively. Consistently detectable miRNAs that were significantly different after HTO were chosen for further validation of the preoperative and postoperative SF samples obtained from 22 patients. Among the 84 analyzed miRNAs, 14 miRNAs were detected in the SF of 6 patients, with 3 of these miRNAs, miR-30a-5p, miR-29a-3p and miR-30c-5p, showing significant fold changes after HTO.

miR-30a-5p has been previously implicated in the progression of lung [21], colorectal [22], and other types of cancers. Previously, the miR-30a-5p family has been found to be involved in the cardiovascular pathophysiology that leads to myocardial infarction [23, 24]. miR-30a-5p is also associated with rheumatoid arthritis and inflammation in septic conditions [25, 26]. Shen et al. reported that miR-30a-5p is highly expressed in the cartilage of patients with osteoarthritis, and its high expression can block chondrocytes, which induces the apoptosis of chondrocytes by targeting protein kinase B [27]. However, Caserta et al. noted that circular miR-30a-5p is inversely related to pro-inflammatory mediators, such as interleukin-1β and interleukin-6 [26]. In our study, miR-30a-5p was highly expressed in the preoperative SF samples of patients with OA and showed significant changes at the 6-month follow-up appointment, but no significant change was found in the validation.

Studies have shown the association between miR-29a-3p and ankylosing spondylitis (AS) [28, 29]. Although data on miR-29a-3p levels in AS are inconsistent, Prajzlerova et al. reported that patients with advanced-stage AS with extensive bone formation have higher levels of TGF-β, as well as miR-29a suppression and increased bone formation [30]. In addition, Sox9 represses the expression of miR-29a-3p in chondrocytes [31]. In our study, miR-29a-3p significantly increased after HTO, which is consistent with these previous investigations. miR-30c-5p has mainly been reported in relation to cancers [32, 33]; however, studies on chronic inflammatory disease have shown its involvement in endothelial dysfunction [34] and macrophage-mediated inflammation [35]. miR-30c-5p suppresses inflammatory pathways and is involved in related organs. Members of the miR-30 family are associated with the pathology of human OA, as highlighted in a previous study [36]. Moreover, Kung et al. showed that miR-30c-5p is an important potential therapeutic target in OA owing to its role in the disease progression and it can be targeted throughout a wide therapeutic window to reduce the pathogenic development of OA. In their study, miR-30c-5p was significantly down-regulated in the mouse OA model, and *Npr3*, an experimentally validated therapeutic target, was confirmed by bioinformatics analysis [37]. In our study, since miR-30c-5p was found to regulate OA, our findings are consistent with the results of previous studies. The correlations of the 3 miRNAs evaluated in our study were inconsistent with those reported in previous studies. Nonetheless, these miRNAs can be considered common factors in the inflammatory response related to OA of the knee; however, in this study, we focused on SF samples. Thus, it is possible that the pathogenesis of regulation could be different from the processes examined in previous studies.

Along with the 3 identified miRNAs, 4 additional previously reported miRNAs were validated in 22 patients. The changes in clinical symptoms and radiological measurements were used to identify specific miRNAs related to improved outcomes. The 4 miRNAs added in the validation phase were identified in circulating miRNA signatures in OA knee SF samples [38, 39]. These are known to be circulating miRNAs that are differentially expressed in OA. Among the 7 miRNAs, 1 from the profiling stage, miR-30c-5p, and 3 added at the validation stage, miR-378a-5p, miR-23a-3p and miR-27b-3p, showed significant changes. Therefore, our results were consistent with those of previous OA studies that used SF samples, except for miR-140-3p, which showed a difference after HTO, albeit statistical significance was not observed. Furthermore, Yang et al. also reported that the expression of miR-140 in SF was significantly reduced in patients with OA [40]. After Bonferroni correction, however, only 2 miRNAs showed significant differences: miR-30c-5p from the profiling phase and miR-23a-3p from the validation phase. A previous study showed that IL-1β contributes to the release of miR-23a-3p in the SF [38]; however, our patients had a K-L grade of II or III. Hence, this finding is different from that of our present study on the progression of OA. miR-378a-5p is highly expressed in late OA, and miR-27b-3p also shows a high fold change in late OA/early OA. Therefore, these two miRNAs may not have shown any significant difference in our study for patients with early and late medial compartment knee OA [38]. miR-30c-5p has not been evaluated in OA, but it might be an important SF miRNA in medial compartment knee OA, as previously noted in this study.

For clinical relevance, possible correlations between improvements in radiological and functional outcomes and the levels of 7 miRNAs were analyzed. None of the 7 miRNAs was correlated with age, gender, or body mass index data. Four miRNAs correlated with the postoperative VAS score; however, only miR-30c-5p showed significant fold changes that correlated with the postoperative VAS score. The subjective nature of pain and inconsistencies observed during the follow-up may explain this result. Nevertheless, the importance of pain as an outcome of clinical evaluation cannot be disregarded [41]. Moreover, these clinical correlations were only measured with 22 patients, so an evaluation with a larger cohort should also be considered. These results do not definitively prove an association between SF miRNAs and HTO in patients with medial compartment knee OA; however, based on the data presented in this study, we hypothesize that differences in miRNA expression in SF reflect miRNA changes at medial compartment knee OA sites and are related to the inflammatory process.

There are some limitations to our study. In the profiling phase, we can use only 84 miRNAs panel, so 84 miRNAs were screened. To compensate for this, several important miRNAs reported to be related to the progression of osteoarthritis on literature were added in the validation phase. Further studies involving larger patient cohorts are needed to confirm the findings presented in this study, as only a small proportion of the SF miRNAs has been analyzed. Additionally, there was no positive control in our study. A positive control may be needed as the change of each miRNA in the SF would be a result of surgical intervention and not entirely specific to HTO. To account for this, we not only compared preoperative and postoperative expression of miRNAs but also compared clinical and radiologic improvements specific to HTO. Finally, this study focused on miRNAs related to the improvement of OA after HTO intervention; however, the underlying mechanisms between miRNAs and post-HTO prognosis were not evaluated. This study provides fundamental basic research outputs to identify potential prognostic miRNAs based on the change of miRNA’s expressions after HTO in medial compartmental knee OA. Further investigations are required to determine clinical implications of the change of these miRNAs’ expressions after HTO.

Biomarkers are used to evaluate OA improvement in patients in addition to clinical symptoms and radiologic assessments. To the best of our knowledge, this study is the first to screen miRNAs in the SF from patients with medial compartment knee OA and to identify a panel of SF circulating miRNAs that can help evaluate the improvement of OA between the preoperative and postoperative periods. Previous studies have focused on the regeneration of cartilage after HTO with mechanical loading changes. In this study, the screening identified 2 miRNAs, miR-30c-5p and miR-23a-3p, that were significantly altered after HTO. Moreover, miR-30c-5p showed a significant correlation with the postoperative pain score. A direct relationship between miRNA expression and clinical improvement in patients was not definitively demonstrated by our results. Nonetheless, our results do suggest the potential of these miRNAs as biomarkers to indicate clinical improvement after HTO.

## Supporting information

S1 Table Spearman’s rank correlation coefficients between the expression of 7 miRNAs normalized at the validation stage and outcome factors. (DOCX)
