# Supplementary material for: Significant changes in synovial fluid microRNAs after high tibial osteotomy in medial compartmental knee osteoarthritis: Identification of potential prognostic biomarkers
Source: PLoS One. 2020 Jan 10;15(1):e0227596. doi: 10.1371/journal.pone.0227596 (PMC6954069; doi:10.1371/journal.pone.0227596)
Supplement: S1 Table — (DOCX) [file pone.0227596.s001.docx]

**S1 Table.** **Spearman’s rank correlation coefficients between the expression of 7 miRNAs normalized at the validation stage and outcome factors.**

|  |  | | | **Radiologic outcome** | | | | |
| --- | --- | --- | --- | --- | --- | --- | --- | --- |
|  | **Gender** | **Age** | **BMI** | **K-L grade** | **Preoperative varus** | **Postoperative valgus** | **Correction angle** | **Weight-bearing line** |
| miR-30a-5p | 0.181 | 0.340 | -0.169 | -0.113 | 0.133 | 0.289 | 0.305 | 0.253 |
| miR-29a-3p | 0.252 | 0.228 | -0.222 | -0.182 | -0.061 | 0.216 | 0.089 | 0.182 |
| miR-30c-5p | -0.010 | 0.236 | -0.075 | -0.090 | -0.028 | 0.379 | 0.229 | 0.354 |
| miR-378a-5p | 0.342 | -0.020 | -0.319 | -0.377 | -0.181 | 0.024 | -0.142 | 0.037 |
| miR-27b-3p | 0.033 | 0.204 | -0.163 | -0.246 | -0.023 | 0.422 | 0.251 | 0.390 |
| miR-140-3p | 0.170 | 0.352 | -0.178 | 0.070 | 0.104 | 0.159 | 0.197 | 0.119 |
| miR-23a-3p | -0.084 | 0.103 | -0.236 | -0.404 | -0.015 | 0.235 | 0.142 | 0.208 |

|  | **Clinical outcome** | | | |
| --- | --- | --- | --- | --- |
|  | **Preoperative VAS** | **Preoperative WOMAC total** | **Postoperative VAS** | **Postoperative WOMAC total** |
| miR-30a-5p | -0.023 | -0.219 | **-0.480*** | 0.001 |
| miR-29a-3p | 0.094 | -0.091 | -0.395 | 0.066 |
| miR-30c-5p | 0.077 | -0.048 | **-0.453*** | -0.058 |
| miR-378a-5p | 0.158 | -0.197 | -0.031 | -0.045 |
| miR-27b-3P | -0.040 | -0.267 | **-0.449*** | -0.153 |
| miR-140-3P | -0.016 | -0.069 | **-0.489*** | 0.039 |
| miR-23a-3p | -0.101 | -0.185 | -0.293 | -0.077 |

*p-value < 0.05
